# D409H GBA1 mutation accelerates the progression of pathology in A53T α-synuclein transgenic mouse model

**DOI:** 10.1186/s40478-018-0538-9

**Published:** 2018-04-27

**Authors:** Donghoon Kim, Heehong Hwang, Seulah Choi, Sang Ho Kwon, Suhyun Lee, Jae Hong Park, SangMin Kim, Han Seok Ko

**Affiliations:** 1 Neuroregeneration and Stem Cell Programs, Institute for Cell Engineering, The Johns Hopkins University School of Medicine, Baltimore, MD 21205 USA; 2 Department of Neurology, The Johns Hopkins University School of Medicine, Baltimore, MD 21205 USA; 3 Diana Helis Henry Medical Research Foundation, New Orleans, Louisiana 70130 USA; 4 Adrienne Helis Malvin Medical Research Foundation, New Orleans, Louisiana 70130 USA

**Keywords:** Parkinson’s disease, Gaucher’s disease, Glucocerebrosidase 1, D409H GBA1 mutation, α-synuclein

## Abstract

Heterozygous mutations in glucocerebrosidase 1 (*GBA1*) are a major genetic risk factor for Parkinson’s disease and Dementia with Lewy bodies. Mutations in *GBA1* leads to GBA1 enzyme deficiency, and GBA1-associated parkinsonism has an earlier age of onset and more progressive parkinsonism. To investigate a potential influence of GBA1 deficiency caused by mutations in *GBA1* on the disease progression of PD, GBA1 mice carrying D409H knock-in mutation were crossbred with the human A53T (hA53T) α-synuclein transgenic mice. Here, we show that GBA1 enzyme activity plays a significant role in the hA53T α-synuclein induced α-synucleinopathy. The expression of D409H GBA1 markedly shortens the lifespan of hA53T α-synuclein transgenic mice. Moreover, D409H GBA1 expression exacerbates the formation of insoluble aggregates of α-synuclein, glial activation, neuronal degeneration, and motor abnormalities in the hA53T α-synuclein transgenic mice. Interestingly, the expression of D409H GBA1 results in the loss of dopaminergic neurons in the substantia nigra pars compacta of hA53T transgenic mice. Taken together, these results indicate that GBA1 deficiency due to D409H mutation affects the disease onset and course in hA53T α-synuclein transgenic mice. Therefore, strategies aimed to maintain GBA1 enzyme activity could be employed to develop an effective novel therapy for GBA1 linked-PD and related α-synucleinopathies.

## Introduction

Parkinson’s Disease (PD) is a neurodegenerative disorder that affects approximately 1-2% of the elderly population [33]. Common characteristics of PD include the selective loss of dopaminergic neurons and the formation of Lewy bodies (LBs) and Lewy neurites (LNs) in surviving neurons in the substantia nigra pars compacta (SNpc) and locus coeruleus (LC), which eventually result in motor impairment [39]. Many pathologically sequestered protein aggregates are found in LBs in which α-synuclein is a dominating component [43]. Despite the remaining mystery of its exact function of α-synuclein, it has been known to foster neurodegeneration in several diseases such as PD, Dementia with Lewy Bodies (DLBs) and multiple system atrophy (MSA) [13].

Lysosomal glucocerebrosidase 1 (GBA1) enzyme catalyzes the breakdown of glycosylceramide into ceramide and glucose [18]. Homozygous mutations in *GBA1* cause a lysosomal storage disorder, Gaucher disease, whereas heterozygous mutations in *GBA1* are implicated in PD and DLB [9, 12, 42]. Mutations in *GBA1* lead to GBA1 enzyme deficiency and result in α-synuclein accumulation [27, 41]. Clinical pathology of PD, in which *GBA1* mutations are present, displayed the presence of a greater number of LBs and LNs [4, 25]. Recent studies have revealed that GBA1 enzyme activity and the steady-state level of wild type GBA1 protein are both reduced in the postmortem of PD patients with and without *GBA1* mutations [1, 2, 11, 31, 36], indicating the pivotal role of GBA1 on the development of sporadic PD.

To date, the relationship between Gaucher disease and α-synucleinopathies such as PD, DLBs, and MSA has been unraveled to some extent such that PD and LBDs patients with *GBA1* mutations typically show an earlier onset of the diseases and more severe symptoms than control group [32]. In addition, the early-onset PD was identified in the patients with low GBA1 enzyme activity through an imaging study [21]. Several studies attempted to define the effect of GBA1 deficiency on α-synuclein accumulation, turn over and its consequent pathology in vivo [8, 10, 30, 37, 44]. Although the studies have partially relationship among GBA1, α-synuclein, and PD, the animal models fail to represent GBA1-associated Parkinsonism, lacking an earlier age of PD onset and dopaminergic neurodegeneration. To investigate the linkage between GBA1 deficiency and PD, we crossbred GBA1 mice harboring D409H knock-in mutation with human A53T α-synuclein transgenic (Tg) mice. These mice have exhibited severe motor impairments and neuropathology accompanying typical alpha-synuclein pathology including serine 129 phosphorylation, the formation of alpha-synuclein fibrils and truncated alpha-synuclein, as well as biochemical defects including mitochondrial defects and endoplasmic reticulum stress, but there are no obvious neuropathological changes in the SNpc region [5, 6, 19, 24]. Using these mice, we assessed the effects of D409H GBA1 mutation on the major phenotypes such as neurodegeneration, accumulation of α-synuclein aggregates, endoplasmic reticulum (ER) stress, and neuroinflammation as well as shortened lifespan were all observed in the A53T α-synuclein Tg mice with the disease onset. Notably, the expression of D409H GBA1 mutation in the A53T α-synuclein Tg mice accelerated the PD progression.

## Materials and methods

### Animals

All experimental procedures were followed according to the guidelines of Laboratory Animal Manual of the National Institute of Health Guide to the Care and Use of Animals, which were approved by the Johns Hopkins Medical Institute Animal Care and Use Committee. GBA1 D409H knock-in (KI) mice were kindly provided by Dr. Gregory A. Grabowski [45] and human alpha-Syn (A53T) transgenic mice were purchased at the Jackson Lab (Stock#: 006823). The mice were back-crossed with C57BL/6 mice (Jackson Lab), and human A53T α-synuclein Tg mice with D409H GBA1 knock-in mice were generated for the present study.

### Stereological assessment

For stereological assessment [47], mice were perfused with PBS followed by 4% paraformaldehyde. After post-fixed with 4% paraformaldehyde for 12 h, the tissue samples were cryoprotected with 30% sucrose, and processed for immunohistochemistry. 50 μm coronal sections were cut throughout the brain including substantia nigra and every 4th section was used for analysis. The rabbit polyclonal anti-TH (1:1000; Novus) was incubated in blocking solution. The signals were visualized using DAB kit (Vector Laboratories) followed by incubation with biotinylated secondary antibodies and streptavidin-conjugated horseradish peroxidase (HRP) (Vectastain ABC kit, Vector Laboratories). The stained tissue sections were mounted onto slides and counterstained with thionin for Nissl substance. The total number of TH-, and Nissl-positive neurons in the SNpc was counted using Optical Fractionator probe of Stereo Investigator software (MicroBrightfield).

### Immunostaining

α-synuclein pathology in the brainstem and the SNpc region were visualized by staining with anti-pS^129^ antibody (1:1000; Abcam). The semi-quantitative grading of p-α-Syn pathology of the SNpc was quantified as previously described [16] with minor modification. The samples were graded using a 0-3 semi-quantitative density scale.

Microglia and astrocyte were stained with anti-Iba-1 (1:1000; Wako) or anti-GFAP (1:2000; Dako), antibodies followed by incubation with biotin-conjugated anti-rabbit antibody and ABC reagents (Vector Laboratories). Then, sections were developed using SigmaFast DAB Peroxidase Substrate (Sigma-Aldrich, St. Louis, MO, USA). The number of microglia and densities of astrocyte in the SNpc region were measured using ImageJ software. The GlcCer-positive signals were stained with anti-GlcCer antibody (1:500, Glycobiotech), followed by incubation with CY3-conjugated anti-donkey secondary antibody. The fluorescent images were acquired through a Zeiss confocal microscope (LSM 710, Zeiss Confocal).

### Western blotting

The ventral midbrain tissues were dissected and prepared in lysis buffer that consist of 10 mM Tris-HCL, 150 mM NaCl, 5 mM EDTA, 0.5% Nonidet P-40, 10 mM Na-β-glycerophosphate, Phosphate inhibitor mixture I and II (Sigma-Aldrich, St. Louis, MO, USA), and complete protease inhibitor mixture (Roche) at pH 7.4. Then the tissues were homogenized using a Diax 900 homogenizer (Sigma-Aldrich, St. Louis, MO, USA). After homogenization, samples were centrifuged at 12000 × g for 20 min, supernatants were collected, and protein levels of each supernatant were quantified. Electrophoresis on 8-16% gradient SDS-PAGE was performed in order to resolve the 20 μg of proteins from the ventral midbrain tissues. The proteins were then transferred to nitrocellulose membranes. The membranes were blocked with blocking solution (Tris-buffered saline containing 5% non-fat dry milk and 0.1% Tween-20) for 1 h and incubated at 4 °C overnight with anti-α-synuclein (1:1000; Sigma S5566), anti-α-synuclein (1:1000; BD Biosciences), or anti-grp78 (1:500; Santa Cruz; sc-1050) antibodies, followed by HRP-conjugated secondary antibody (1:5000; GE Healthcare) for 1 h at RT. Finally, the membranes were re-probed with HRP-conjugated β-actin antibody (1:50,000; Sigma-Aldrich, St. Louis, MO, USA) after the blots were stripped.

### GBA1 enzyme (GCase) activity assay

The GCase activity assay has been performed as previously described [3, 27]. Mouse ventral midbrain tissues were homogenized in the buffer containing 0.25 M sucrose, 10 mM HEPES (pH 7.4) and 0.1 M EDTA, centrifuged at 6800 × g, 4 °C, for 5 min, and the supernatant was collected. The supernatant was centrifuged at 17,000 × g for 10 min, and the pellet enriched with lysosomes was collected in 50 μl of activity assay buffer 0.25% Triton X-100 (Sigma-Aldrich), 0.25% Taurocholic acid (Sigma-Aldrich), 1 mM EDTA, in citrate/phosphate buffer, pH 5.4. The GCase activity was measured by adding 50 μl of 1% BSA, adding 1 mM 4-Methylumbelliferyl β-glucophyranoside (4-MU; M3633, Sigma-Aldrich) and/or 10 mM conduritol B epoxide (CBE, Sigma-Aldrich). The samples were incubated for 40 min at 37 °C, followed by the addition of 50 μl (equi-volume) of 1 M glycine at pH of 12.5 to terminate the reaction. Sample volume of 100 μL per well was prepared on 96 well plate (Nunc, # 136101). The fluorescence was measured via a Perkin Elmer plate reader (ex = 355 nm, em = 460 nm, 0.1 s). GCase1 activity was obtained by subtracting the GCase activity in presence of CBE from the total GCase activity of each sample. 95-97% of GCase activity was reduced by CBE treatment.

### Dot-blot assay

Samples were loaded onto the pre-wetted nitrocellulose membrane using Bio-Dot microfiltration apparatus (Bio-rad). After washing each sample with tris-buffered saline, samples were blocked with 5% non-fat dry milk in tris-buffered saline containing 0.1% tween-20. Membranes were incubated with anti-α-synuclein filament antibody (1:1000; Abcam) or GlcCer antibody (1:500, Glycobiotech) at 4 °C overnight, followed by HRP-conjugated rabbit secondary antibody (GE Healthcare) for 1 h at RT.

### Behavioral test

For the pole test [47], the mice were trained for two consecutive days before the actual test. Each training session consisted of three test trials. Animals were placed on the top of the pole (75 cm of metal rod at diameter of 9 mm) facing the head up direction. The time taken to turn and total time taken to reach the base of the pole were recorded. The maximum cutoff time to stop was 120 s. For the rotarod test [23], the mice were trained for three consecutive days (four 5-min trials, 5-min apart) to acclimate them to the rotarod apparatus. During the test period, mice were placed on the rotarod with increasing speed, from 4 rpm to 40 rpm in 300 s. The latency to fall off was recorded under blind condition to different groups.

### Statistical analysis

Data were presented as mean ± SEM with at least 3 independent experiments. Representative morphological images were taken out of at least 3 experiments with parallel results. An unpaired two-tailed Student’s test or an ANOVA test followed by Bonferroni post hoc analysis was conducted to assess the statistical significance. Assessments with *p* < 0.05 were considered significant.

## Results

### GBA1 enzyme deficiency caused by GBA1 D409H mutation increases the levels of α-synuclein

**Fig. 1:**
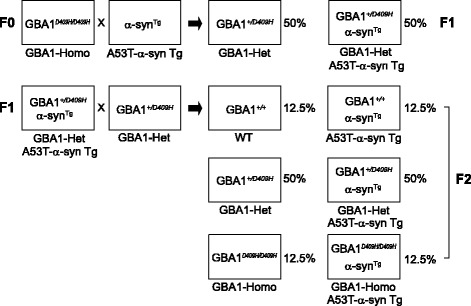
Breeding strategy. To test our hypothesis that decreased GBA1 enzyme activity affects neurodegeneration in human A53T α-synuclein mouse model of PD, the GBA1^D409H/D409H^ knock-in mice were crossbred with the hA53T α-synuclein mice

**Fig. 2:**
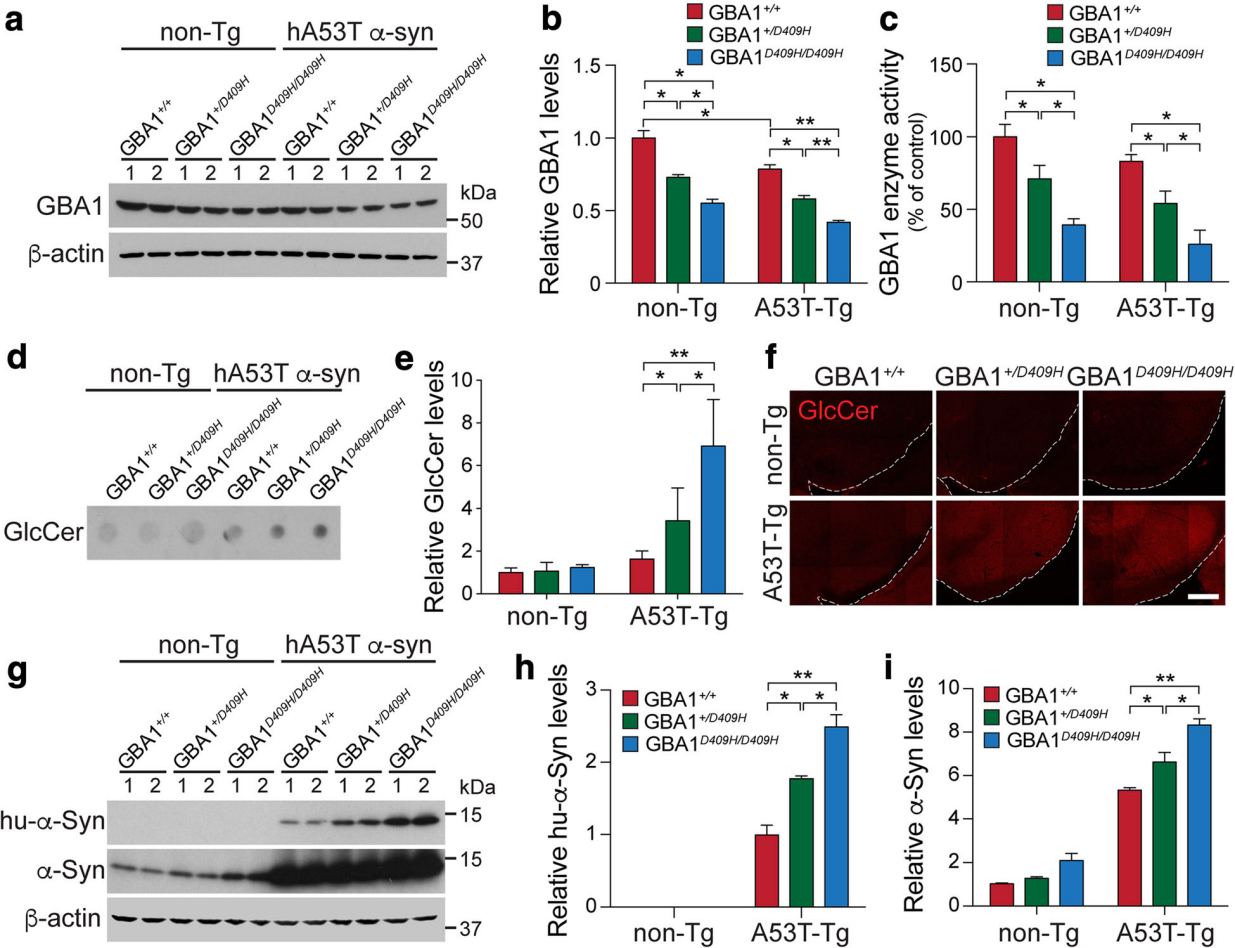
The levels of α-synuclein are dependent on GBA1 enzyme activity. **a** and **b,** The expression levels of GBA1 were quantified in the ventral midbrain of the indicated genotypes at 6 months of age. **c**, GBA1 enzyme activity assay was conducted in the ventral midbrain of the indicated genotypes at 6 months of age. **d,** Dot blot shows relative GlcCer levels in the ventral midbrain of the indicated genotypes at 6 months of age. **e,** The relative levels of GlcCer were quantified. **f,** Representative images of GlcCer by immunostaining in the SN of the indicated genoypes at 6 months of age. The scale bar is 200 μm. **g,** Steady-state levels of human and mouse α-synuclein were monitored in the ventral midbrain of the indicated genotypes via Western blot analysis at 6 months of age. **h** and **i,** The expression levels of human and mouse α-synuclein were quantified. DATA are expressed as mean ± SEM (*n* = 6 for the each group)

To test our hypothesis that decreased GBA1 enzyme activity due to mutation in GBA1 affects neurodegeneration in the hA53T α-synuclein transgenic (Tg) mouse model of PD, the GBA1^D409H/D409H^ mutant mice [45] were crossbred with the hA53T α-synuclein (α-Syn) Tg mice (Fig. 1). GBA1 expression level was reduced to 70% in the ventral midbrain tissues of the GBA1^+/D409H^ mice and to 55% in the ventral midbrain tissues of the GBA1^D409H/D409H^ mice when compared to the wild type mice. GBA1 expression was further reduced to 48% in the hA53T α-Syn;GBA1^+/D409H^ and to 42% in the hA53T α-Syn;GBA1^D409H/D409H^ mice (Fig. 2a and b). GBA1 enzyme activity was reduced to 71% in the brain tissues of the GBA1^+/D409H^ mice and to 39% in the ventral midbrain tissues of the GBA1^D409H/D409H^ mice when compared to the wild type mice. GBA1 enzyme activity was further reduced to 54% in the hA53T α-Syn;GBA1^+/D409H^ and to 25% in the hA53T α-Syn;GBA1^D409H/D409H^ mice (Fig. 2c). Glucosylceramide (GlcCer), a substrate of GBA1, was accumulated by 3.4 folds and 6.9 folds in the hA53T α-Syn;GBA1^+/D409H^ and the hA53T α-Syn;GBA1^D409H/D409H^ mice, respectively (Fig. 2d and e). Similar result was observed in the SN tissues as assessed by GlcCer immunofluorescence staining (Fig. 2f). The levels of overexpressed hA53T α-synuclein were increased by 1.8 folds in the hA53T α-Syn;GBA1^+/D409H^ and by 2.5 folds in the hA53T α-Syn;GBA1^D409H/D409H^ mice at 6 months of age (Fig. 2g and h). The levels of total α-synuclein expression (endogenous mouse α-synuclein and overexpressed hA53T α-synuclein) were increased by 6.6 folds in the hA53T α-Syn;GBA1^+/D409H^ and by 8.3 folds in the hA53T α-Syn;GBA1^D409H/D409H^ mice at 6 months of age compared with non-Tg mice. Additionally, we found that the levels of endogenous mouse α-synuclein are increased in the dependent manner of GBA1 enzyme activity in GBA1 mutant mice (Fig. 2i). Thus, the steady-state levels of both endogenous α-synuclein and hA53T α-synuclein are dependent on the enzyme activity of GBA1 resulting from D409H mutation.

### D409H GBA1 expression shortens lifespan and leads to dopaminergic degeneration in hA53T α-synuclein Tg mice

**Fig. 3:**
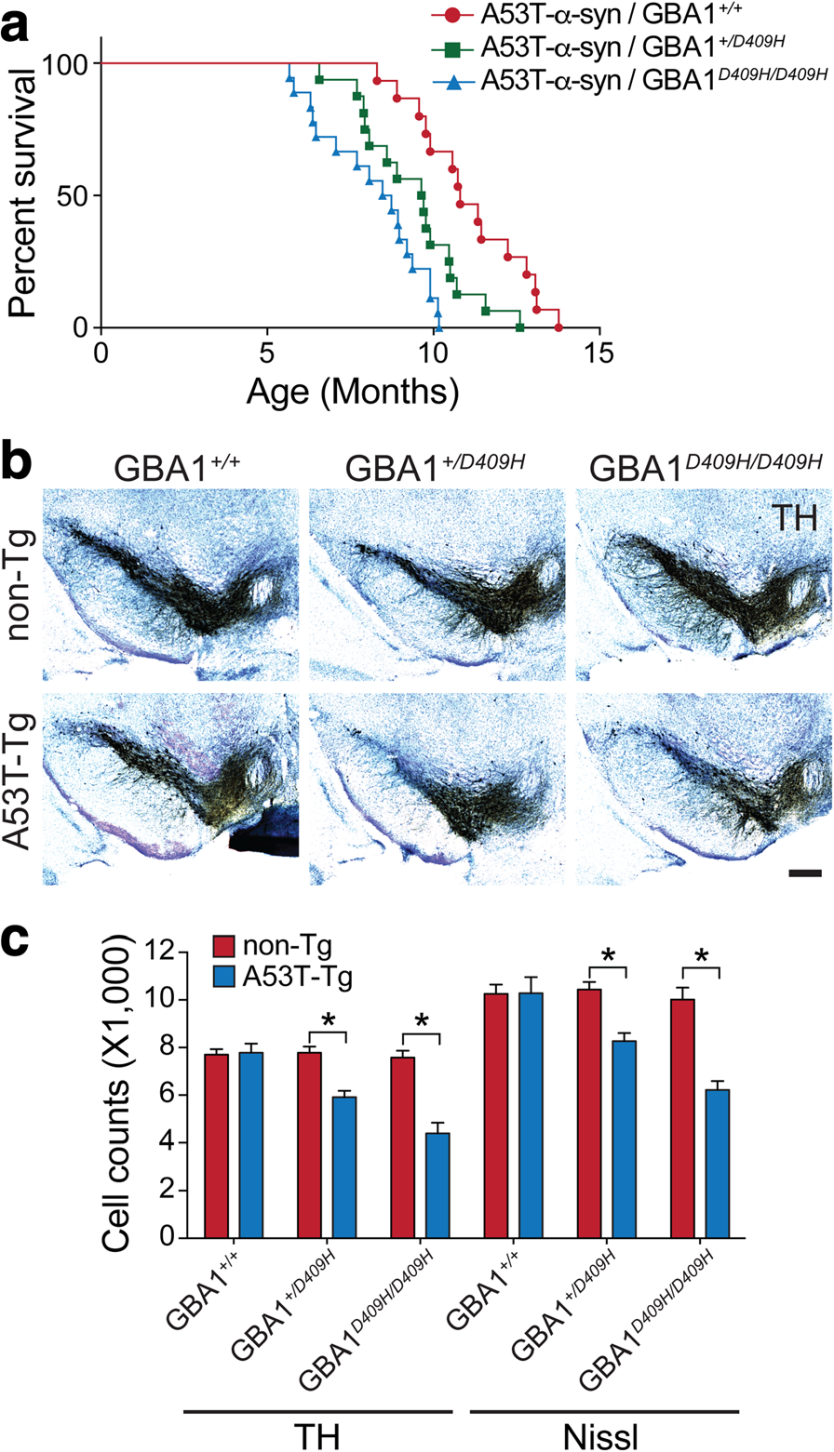
D409H GBA1 expression shortens lifespan and leads to dopaminergic degeneration in the hA53T α-synuclein transgenic mice. **a*,*** Survival was monitored from littermates with the following genotypes: hA53Ta-Syn/GBA1^*+/+*^ (*n* = 15), hA53T α-Syn/GBA1^*+/D409H*^(*n* = 16), hA53T α-Syn/GBA1^*D409H/D409H*^ (*n* = 18) mice. GBA1 mutation induces the lethal phenotype and TH-positive neuronal loss in the SNpc of A53T mutant α-synuclein transgenic mice. **b** and **c*,*** The number of TH-positive neurons in the SNpc was counted using stereological analysis with the indicated genotypes at 6 months of age. The scale bar is 200 μm. DATA are expressed as mean ± SEM (*n* = 6 for the each group)

The hA53T mutant α-synuclein Tg mice develop adult-onset phenotypes with rapidly progressive motor impairment that eventually leads to death [19]. To examine whether D409H GBA1 expression bearing decreased enzyme activity affects the lifespan of hA53T α-synuclein Tg mice, littermates with the following genotypes were separated and aged: hA53T α-Syn, hA53T α-Syn;GBA1^+/D409H^, hA53T α-Syn;GBA1^D409H/D409H^, and their survival was monitored (Fig. 3a). The hA53T α-synuclein Tg mice lived an average of 10.8 months, as previously described [7]. The hA53T α-Syn;GBA1^+/D409H^ lived an average of 9.7 months and the hA53T α-Syn;GBA1^D409H/D409H^ lived an average of 8.6 months, indicating that the hA53T α-Syn;GBA1^D409H/D409H^ significantly shortens lifespan of the hA53T α-synuclein Tg mice by 2.2 months. Therefore, decreased enzyme activity due to D409H GBA1 expression has a boosting impact that expedites the onset and progression of the lethal phenotype induced by α-synuclein pathologies in the hA53T α-synuclein Tg mice.

To determine whether the reduced GBA1 enzyme activity induces the loss of dopaminergic neurons in the hA53T α-synuclein mice, the number of TH positive neurons in the SNpc was counted via an unbiased stereological analysis of the genotypes at 6 months of age (Fig. 3b and c). As previously described, at 6 months of age when the mice are asymptomatic, there was no obvious dopaminergic neurodegeneration in the hA53T α-synuclein Tg mice. In contrast, there was an approximately 24% loss of dopaminergic neurons in the SNpc of the hA53T α-Syn;GBA1^+/D409H^. Furthermore, there was 43% neuronal loss in the SNpc of the hA53T α-Syn;GBA1^D409H/D409H^ mutant mice.

### D409H GBA1 expression leads to reduction of the dopaminergic fiber density and alters behavioral deficits in the A53T α-synuclein Tg mouse models

**Fig. 4:**
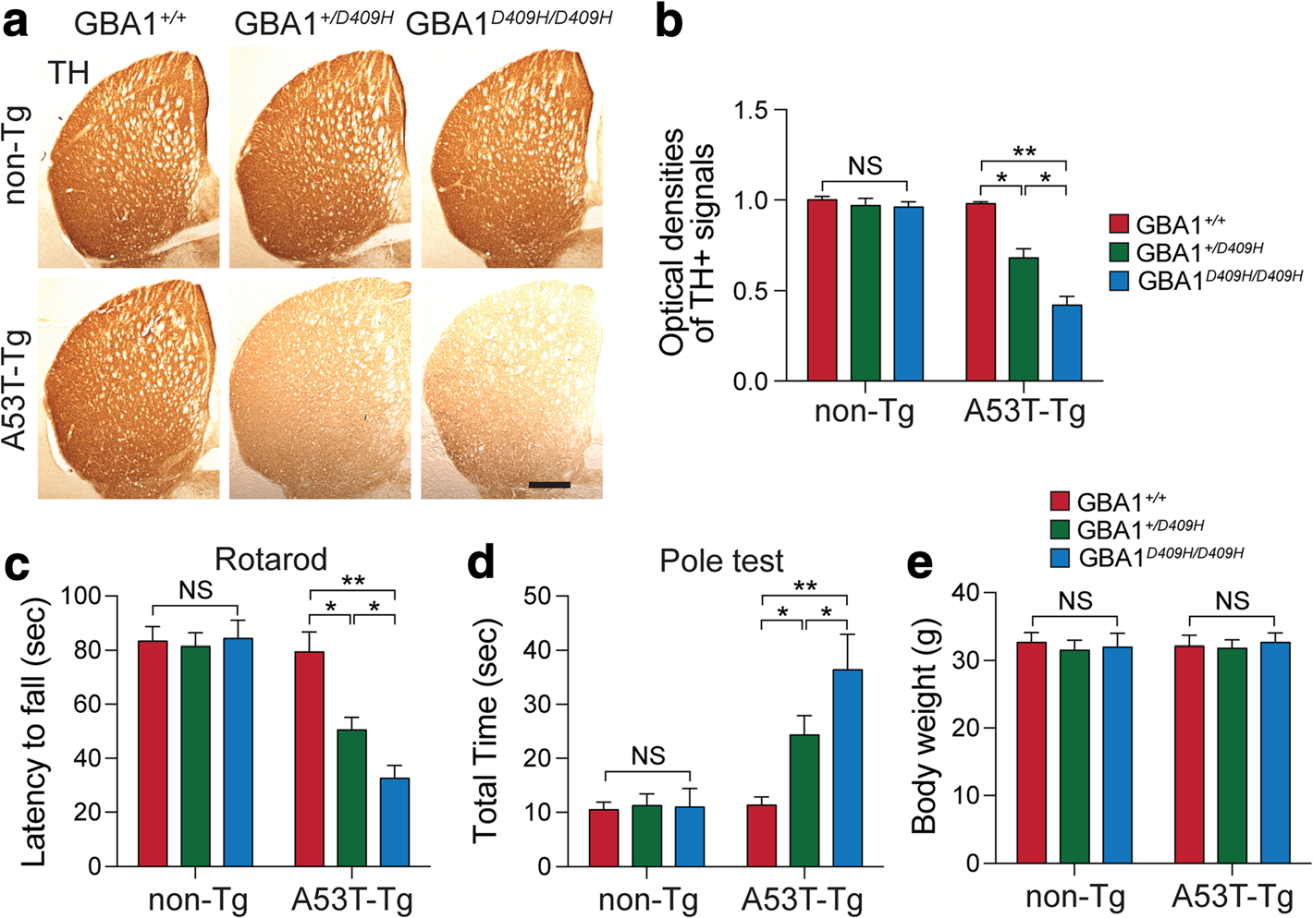
D409H GBA1 expression promotes reduction of dopaminergic fiber densities and motor deficit in the striatum of hA53T α-synuclein transgenic mice. **a*,*** The striatal TH-immunopositive fiber density was assessed in the striatum of the indicated genotypes at 6 months of age (*n* = 6 per each group). The scale bar is 250 μm. **b*,*** The optical densities of TH positive signals were quantified. **c** and **d**, Rotarod and Pole test were assessed at 6 months of age with the indicated genotypes (*n* = 7 per each group). e, the body weight of mice were measured with the indicated genotypes (*n* = 15 per each group). DATA are expressed as mean ± SEM

Since D409H GBA1 expression leads to dopaminergic neurodegeneration in the SNpc, next, tyrosine hydroxylase (TH)-immunopositive fiber density in the striatum was assessed (Fig. 4a). At 6 months of age when the mice were asymptomatic, there was no obvious dopaminergic terminal loss in the hA53T α-synuclein mice. However, there was approximately 32% TH fibers were lost in the striatum of the hA53T α-syn;GBA1^+/D409H^ and 58% in the striatum of the hA53T α-Syn;GBA1^D409H/D409H^ compared to the control group (Fig. 4b).

To determine whether D409H GBA1 expression leading to decreased GBA1 enzyme activity leads to the abnormal behavior in the A53T α-synuclein, we performed a pole test, and rotarod analysis using a cohort of 6 months of age of different genotypes (Fig. 4c and d). At 6 months of age, when the hA53T α-synuclein mice were asymptomatic, there was no significant behavioral impairment on the rotarod test. Average latency to fall in the accelerating rotarod was reduced in the hA53T α-Syn;GBA1^+/D409H^. The reduction in latency times was greater in the hA53T α-Syn;GBA1^D409H/D409H^ mice at the 6 months of age (Fig. 4c). We also conducted the pole test since it is a useful method for evaluating the mouse movement disorder caused by striatal dopamine depletion [26]. The pole test revealed that there was a moderate increase in the time to reach to the base of the pole in the hA53T α-Syn;GBA1^+/D409H^ and a significantly greater increase in the hA53T α-Syn;GBA1^D409H/D409H^ at 6 months of age (Fig. 4d). At 6 months of age, however, there was no significant difference in body weight (Fig. 4e).

### D409H GBA1 expression accelerates the accumulation of insoluble α-synuclein species in the brainstem and SNpc of A53T α-synuclein Tg mice

**Fig. 5:**
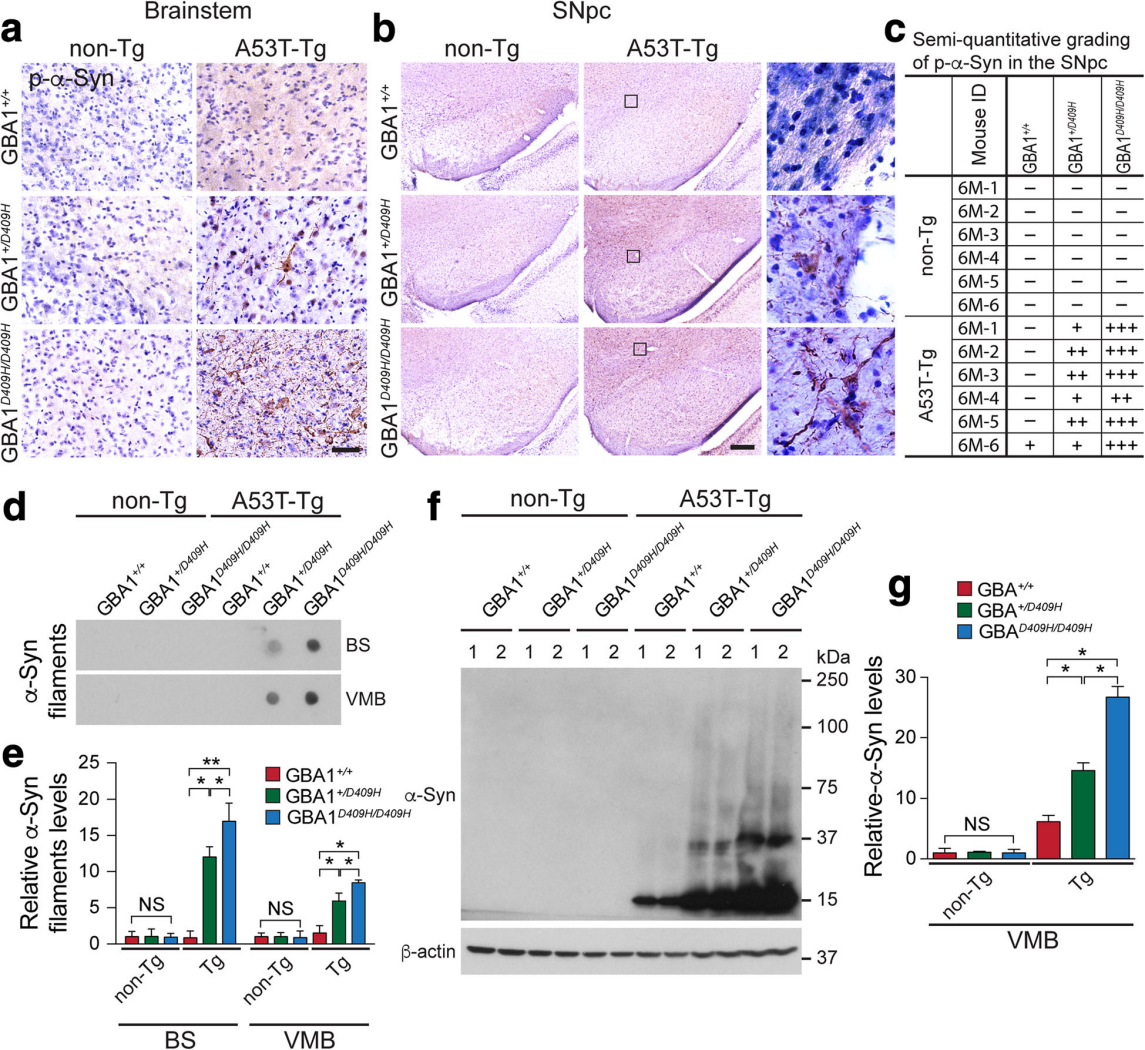
D409H GBA1 expression leads to the accumulation of pathologic α-synuclein aggregates in the brainstem and SNpc of A53T α-synuclein transgenic mice. **a*,*** Representative images of p-α-Syn positive signals in the brainstem . Immunohistochemistry with phosphor-S^129^ α-synuclein (p-α-Syn) antibody was conducted in the lateral vestibular nucleus of the brainstem of the indicated genotypes at 6 months of age. The scale bar represents 50 μm. **b,** Representative images of p-α-Syn signals in the SNpc of the indicated genotypes at 6 months of age at low-magnification and high-magnification. The scale bar is 200 μm. **c,** Semi-quantitative grading of p-α-Syn pathology in the indicated genotypes. **d** and **e*,*** Dot-blot analysis of brainstem (BS) and ventral midbrain (VMB) of bigenic mice (*n* = 3) with α-synuclein filament antibody was performed and quantified. **f** and **g*,*** Accumulation of high molecular weight species of α-synuclein in the ventral midbrain was assessed in the VMB. The ventral midbrain lysates were immunoblotted with α-Syn antibody. The high molecular weight species were quantified

As the accumulation of insoluble high molecular weight species of α-synuclein is a prominent indicator of pathology in the A53T α-synuclein mice [19], it was assessed via various techniques such as immunohistochemistry, dot blot, and immunoblot analysis (Fig. 5). Immunohistochemistry was conducted at 6 months of age when the mice were asymptomatic. At this time point, there was no obvious accumulation of α-synuclein phosphorylated at serine 129, which is closely associated with α-synuclein aggregation, in the SNpc and brainstem of the hA53T α-Syn mice. In contrast, immunohistochemistry revealed that some accumulation of α-synuclein phosphorylated at serine 129 in the brainstem and SNpc of the hA53T α-Syn;GBA1^+/D409H^ and significant accumulation in the brainstem and SNpc of the hA53T α-Syn;GBA1^D409H/D409H^ at 6 months of age (Fig. 5a, b, and c). None of the aggregates was detected in the brainstem and SNpc of non-Tg mice. Dot blot (Fig. 5d, and e) and immunoblot analysis (Fig. 5f and g) also demonstrated that the detergent-insoluble high molecular weight species of α-synuclein accumulated in the ventral midbrain of the hA53T α-Syn;GBA1^+/D409H^ and the accumulation was significantly increased in the ventral midbrain of the hA53T α-Syn;GBA1^D409H/D409H^ at 6 months of age (Fig. 5d, e, f, and g).

### D409H GBA1 expression shows early neuroinflammation in the A53T α-synuclein Tg mice

**Fig. 6:**
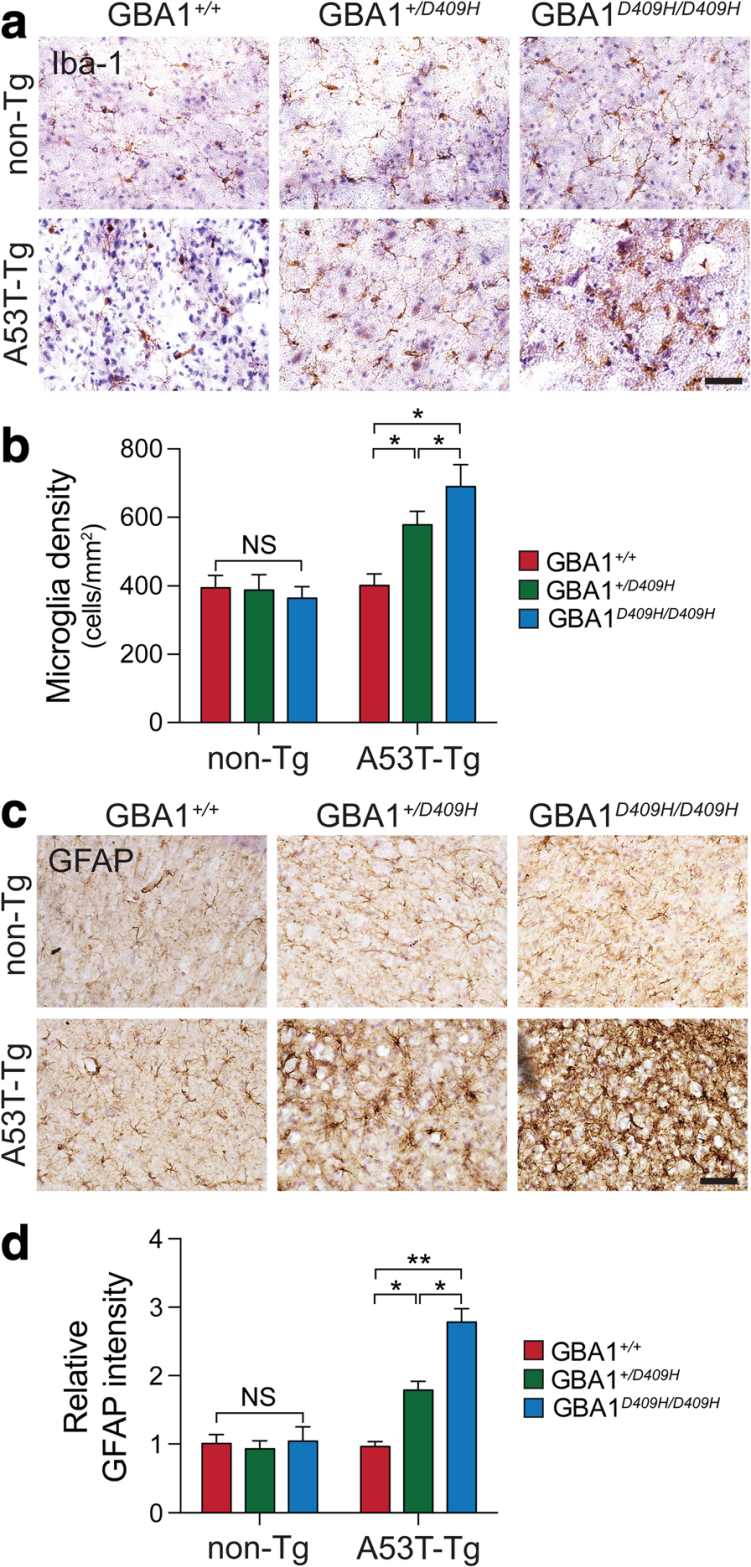
D409H GBA1 expression shows the activation of pathology-associated microglia and astrocyte activation in the SNpc of A53T α-synuclein transgenic mice. **a*,*** Iba-1 immunoreactive microglia were observed in the SNpc region of the indicated genotypes at 6 months of age. The scale bar is 50 μm. **b*,*** The number of microglia was counted. **c*,*** GFAP immunoreactive astrocytes were observed in the SNpc region of the indicated genotypes at 6 months of age. The scale bar is 50 μm. **d*,*** The signals were measured using ImageJ

At 6 months of age there was no significant accumulation of Iba-1 and GFAP in the SNpc of A53T α-synuclein mice. For microglia activation, characterized by the increased expression of Iba-1, serves as an indirect indicator of neuronal abnormality in the A53T α-synuclein Tg mice. Thus, the enhanced expression of Iba1 was determined by immunohistochemistry. There was an increased Iba-1 immunoreactivity in the SNpc of hA53T α-Syn;GBA1^+/D409H^. The immunoreactivity was dramatically increased in the SNpc of the hA53T α-Syn;GBA1^D409H/D409H^ at 6 months of age (Fig. 6a and b). As the accumulation of glial fibrillary acidic protein (GFAP) is a prominent pathological indicator in the A53T α-synuclein Tg mice, its accumulation was also assessed via immunohistochemistry. There was an increased GFAP immunoreactivity in the SNpc of hA53T α-Syn;GBA1^+/D409H^. The accumulation was further increased in the SNpc of the hA53T α-Syn;GBA1^D409H/D409H^ at 6 months of age (Fig. 6c and d).

### D409H GBA1 expression triggers ER stress early in the A53T α-synuclein Tg mice

**Fig. 7:**
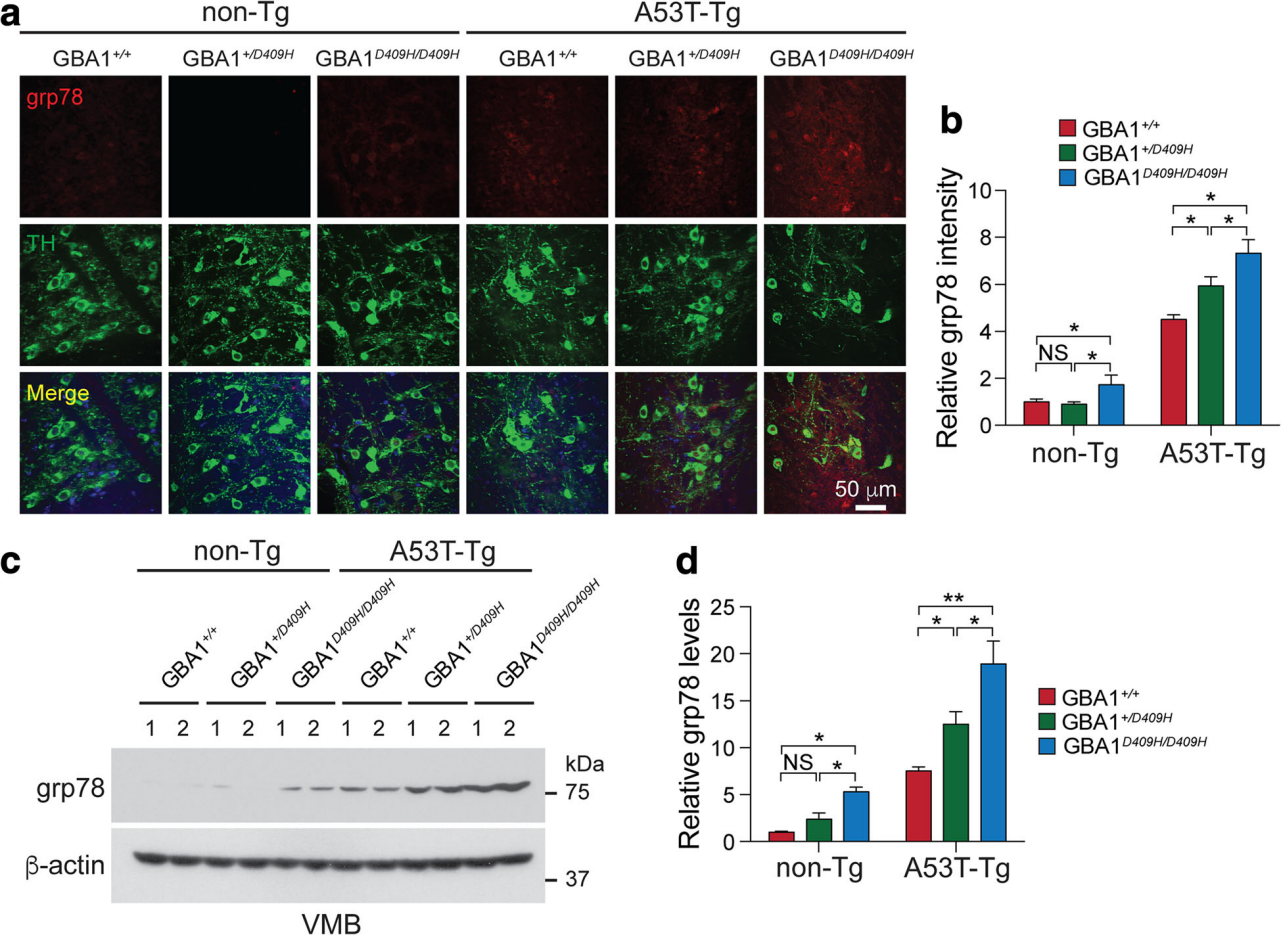
D409H GBA1 expression shows early induction of ER chaperone grp78 in the A53T α-synuclein transgenic mice. **a*,*** Accumulation of grp78, an indicator of ER stress, was analyzed via immunostaining with grp78 and TH antibodies in the SNpc of indicated genotypes at 6 months of age. The scale bar is 50 μm. **b*,*** The fluorescence levels of grp78 were analyzed using Image J and compared to non-Tg (*n* = 3). **c** and **d*,*** Accumulation of grp78 was analyzed via Western blot analysis with grp78 antibody in the ventral midbrain of the indicated genotypes. The grp78 levels were measured using ImageJ

In symptomatic A53T α-synuclein Tg mice there was an accumulation of indicators of ER stress in a number of brain regions, including the brainstem and spinal cord [5]. 78 kDa glucose-regulated protein (grp78/BiP), an indicator of ER stress, was analyzed via immunohistochemisty and Immunoblot analysis using grp78 antibody (Fig. 7). Strikingly, immunohistochemistry demonstrated that there was a moderate increase of grp78 protein level in the SNpc tissues of the hA53T α-Syn;GBA1^+/D409H^ and the upregulation of grp78 was significantly increased in the SNpc tissues of the hA53T α-Syn;GBA1^D409H/D409H^ at 6 months of age (Fig. 7a and c). Immunoblot analysis also demonstrated that the grp78 protein accumulated in the ventral midbrain of the hA53T α-Syn;GBA1^+/D409H^ and the accumulation was further promoted in the ventral midbrain of the hA53T α-Syn;GBA1^D409H/D409H^ at 6 months of age (Fig. 7c and d).

## Discussion

The hypothesis that GBA1 could affect α-synuclein degradation and pathology has been tested in several animal models [8, 10, 30, 37, 44]. These models have allowed us to elucidate the relationship among GBA1, α-synuclein, and PD. However, these animal models do not fully represent the clinical observations seen in the GBA1-associated Parkinsonism such as earlier PD onset and DA neuronal loss. To gain further insights into the mechanisms by which *GBA1* mutations increase the risk for PD and lead to the development of GBA1-assiciated parkinsonism, we crossbred GBA1 mice carrying D409H knock-in mutation with human A53T α-synuclein Tg mice exhibiting neurological abnormalities, accumulation of α-synuclein aggregates, increased ER stress, neuroinflammation, and shortened lifespan [5, 19]. Using this mouse model, we assessed the impact of D409H GBA1 mutation on the major phenotypes of the A53T α-synuclein Tg mice with disease onset and further examined the cardinal features seen in the GBA1-associated Parkinsonism through biochemical and immunohistochemical analyses. Importantly, our findings reveal that the expression of D409H GBA1 mutation resulted in the loss of DA neurons, accelerates disease onset, exacerbates neuroinflammation and ER stress more extensively than the degree seen in the hA53T α-synuclein Tg mice.

It has been reported that mutations in *GBA1* result in the production of misfolded GBA1, increased GBA1 ubiquitination, and premature degradation leading to quantitative loss in the protein levels [22, 46]. We found the reduction of GBA1 protein expression and GBA1 enzyme activity in the brains of GBA1^+/D409H^ and GBA1^D409H/D409H^, which is similar to previous findings that GBA1 deficiency due to expression of L444P mutation or heterozygous *GBA1*-null mutations results in GBA1 enzyme activity deficiency [30]. Importantly, there was even lower GBA1 enzyme activity in the brains of hA53T α-Syn;GBA1^+/D409H^ and hA53T α-Syn;GBA1^D409H/D409H^, which differs from the earlier finding that heterozygous *GBA1*-null mutations in the A53T α-synuclein Tg mice does not lead to GBA1 enzyme deficiency [44]. Although the reason for this discrepancy is unclear, it is conceivable that the GBA1 enzyme deficiency due to D409H mutation may affects the disease onset in A53T α-synuclein Tg mice differently from heterozygous GBA1-null mutations. For instance, the buildup of the misfolded GBA1 D409H mutant protein [29] and α-synuclein aggregates trigger ER stress [5], which would form a positive feedback loop to further impair GBA1 enzyme activity and consequently contribute to α-synuclein pathology and loss of DA neurons in the model. Since it is known that GBA1 enzyme activity was lowest in the SN of PD patients [1, 2, 11, 31], a further investigation will be required to determine GBA1 enzyme activity in different brain regions in this mouse model. In addition, it is possible that GBA1 D409H mutation may affect the lysosomal dysfunction in hA53T α-synuclein Tg, thereby hampering the autophagy/lysosomal degradation of pathological α-synuclein [34]. Future study will be required to test this possibility in our animal model.

In our model, the relationship between GBA1 enzyme activity and α-synuclein accumulation revealed that GBA1 deficiency due to D409H mutation was associated with the increased levels of human and mouse α-synulcein proteins as well as the enhanced levels of high molecular weight α-synuclein aggregates in the ventral midbrain regions. Consistent with previous findings, our observations confirm that decreased GBA1 enzyme activity due to *GBA1* mutations or null leads to increased α-synuclein levels in other models [8, 10, 30] and PD postmortem brains [11, 31]. Since the levels of α-synuclein are greater in the brain of hA53T α-Syn;GBA1^D409H/D409H^ compared to hA53T α-Syn;GBA1^+/D409H^, the accumulation of α-synuclein is dependent on the levels of GBA1 enzyme activity. On the other hand, our study revealed that phosphoserine 129 (pSer) α-synuclein immunoreactivity and high molecular weight α-synuclein species were detected in the ventral midbrain of hA53T α-Syn;GBA1^+/D409H^ and hA53T α-Syn;GBA1^D409H/D409H^ mice at 6 months of age. Moreover, intensities of both pSer α-synuclein immunoreactivity and high molecular weight α-synuclein species were correlated with the enzyme activity levels of GBA1 in the hA53T α-Syn;GBA1^+/D409H^ and hA53T α-Syn;GBA1^D409H/D409H^ mice. However, the pSer immunoreactivity and high molecular weight α-synuclein species were not present in the ventral midbrain of the hA53T α-Syn Tg mice at 6 months of age. Although the underlying mechanism of how D409H expression results in increased pathologic α-synuclein aggregates at the early time point is not clear, it is likely that additional α-synuclein accumulation triggered by GBA1 deficiency due to D409H expression pushes forward the levels of α-synuclein protein to reach quickly the threshold required for pathologic α-synuclein aggregates in the model at 6 months of age, eliciting overt DA neurodegeneration loss in the SNpc and PD related motor deficits in the same model. Based on our current observations, the hA53T α-Syn Tg mouse model may provide a valuable resource to uncover mechanisms of how PD-associated gene mutations can impact PD pathogenesis.

Importantly, we found the loss of nigrostriatal DA neurons in the SNpc of the hA53T α-Syn;GBA1^+/D409H^ and hA53T α-Syn;GBA1^D409H/D409H^, which were not detected in the A53T α-synuclein Tg mice, GBA1^+/D409H^, and GBA1^D409H/D409H^ at 6 months of age. This result has not been reported in the previous studies [10, 37, 44]. One possible explanation for this is due to the accumulation of pathologic α-synuclein aggregates, which may be sufficient to lead to the loss of nigrostriatal DA neurons in the SNpc at the time point. Another explanation might be behind neuroinflammation that contributes to neurodegeneration in neurodegenerative disorders including PD [20, 35, 38]. Neuroinflammation was present in the SNpc of hA53T α-Syn;GBA1^+/D409H^ and hA53T α-Syn;GBA1^D409H/D409H^, but not observed in A53T α-synuclein Tg mice, GBA1^+/D409H^, and GBA1^D409H/D409H^ at 6 months of age. The last explanation for this might be ER stress that contributes to neurodegeneration in neurodegenerative disorders including PD [14, 15, 28]. We also observed significantly changed levels of ER stress in the SNpc of hA53T α-Syn;GBA1^+/D409H^ and hA53T α-Syn;GBA1^D409H/D409H^ at 6 month of age, which were not detected in A53T α-synuclein Tg mice, GBA1^+/D409H^, and GBA1^D409H/D409H^ at the time point.

Although the penetrance of D409H GBA1 mutation is relatively lower than other mutations such as N370S and L444P GBA1 mutations [40], our current findings suggest that GBA1 deficiency due to D409H GBA1 mutation alone is not sufficient to cause PD but additional factors, such as environmental factors or increased levels of α-synuclein could increase the penetrance through rendering the levels of α-synuclein accumulation close to the threshold required for α-synuclein aggregation. Our previous finding that GBA1 deficiency due to L444P GBA1 heterozygous mutation renders DA neurons more susceptible to MPTP intoxication [47] further supports this hypothesis. Our study does not provide a detailed explanation on how D409H GBA1mutation contributes to severe neurodegeneration with the loss of DA neurons in the A53T α-synuclein Tg mice. Further studies need to be undertaken to account for the DA neurodegeneration. Also, it would be interesting to study how the regulation of formation of α-synuclein tetramers and other related multimers as well as the changes in the status of glycosphingolipids (GSLs) in the model is regulated [17].

## Conclusions

In conclusion, our results indicate that GBA1 deficiency due to D409H GBA1 mutation that contributes to α-synuclein accumulation exacerbates neuronal vulnerability in neurodegenerative processes triggered by A53T α-synuclein expression in vivo. The model that recapitulates the cardinal PD phenotypes including loss of DA neurons, LB pathology, and motor deficits can be a useful tool to study in depth the possible mechanisms underlying neurodegeneration due to *GBA1* mutations and to test the efficacy of potential treatment against GBA1-associated PD and Dementia with Lewy bodies (DLB).
